# Editorial: Primary immune regulatory disorders: Coming of age

**DOI:** 10.3389/fped.2023.1155785

**Published:** 2023-03-08

**Authors:** Andrew R. Gennery, Luis I. Gonzalez-Granado, Troy R. Torgerson

**Affiliations:** ^1^ Translational and Clinical Research Institute, Newcastle University, Newcastle upon Tyne, United Kingdom; ^2^ Paediatric Immunology+HSCT, Great North Children’s Hospital, Newcastle upon Tyne, United Kingdom; ^3^ Pediatrics Department, Hospital 12 Octubre, Madrid, Spain; ^4^ School of Medicine, Complutense University, Madrid, Spain; ^5^ Experimental Immunology, Allen Institute for Immunology, Seattle, WA, United States

**Keywords:** primary immune regulatory disorders, interleukin-2 signalling axis, CTLA-4 insufficiency, Ikaros, activated PI3-kinase-δ syndrome, XIAP deficiency, STAT3 gain of function, AIRE deficiency

**Editorial on the Research Topic** Primary immune regulatory disorders: Coming of age

Genetic disorders of the immune system were first recognised as primary immunodeficiencies, characterized by recurrent or persistent infections, often due to so called opportunistic pathogens. The spectrum of infections was wide, including bacterial, viral or fungal infection and affecting, predominantly, the respiratory or gastrointestinal tract. Even in classical primary immunodeficiency disorders, autoimmunity and autoinflammation were a recognized presentation, but infections typically dominated the clinical picture. With the development of accurate genetic profiling, it is now recognized that disorders in genes intimately involved with immune responses may present predominantly with inflammatory or autoimmune features, although infection may still be part of the presentation. These primary immune regulatory disorders (PIRDs) are a recognised and expanding group of inborn errors of immunity, highlighted in this research topic. The disorders that are included represent a mixture of loss-of-function and gain-of-function diseases and demonstrate the heterogenous clinical presentations and severities of PIRDs. Whilst not comprehensive, experts in the field address seven key topics, including genetics, pathogenesis, presentation, diagnosis, and treatment.

Arguably the first PIRD to be described was Immunodysregulation, Polyendocrinopathy, Enteropathy X-linked (IPEX) syndrome. Initially described in the *Scurfy* mouse, it was subsequently recognized in boys who present with a characteristic triad of early onset intractable diarrhea, neonatal type 1 diabetes mellitus and eczema, although other autoimmune features like autoimmune cytopenias are often present (1). The disease is caused by defects in *FOXP3*, encoding the Forkhead Box P3 (FOXP3) transcription factor, critical for normal development and function of regulatory T-lymphocytes (Tregs), required to maintain peripheral tolerance. Other, similar diseases without such mutations are described, and have been described as IPEX-like disorders. A number of these have now been further characterized, and can be considered as disorders of regulatory T-lymphocytes, or Tregopathies. They share a common signaling pathway through Interleukin-2 (IL-2) activation. Consonni et al. give an overview of diseases caused by deficiencies in CD25, STAT5B and FOXP3.

Cytotoxic T-lymphocyte antigen-4 (CTLA-4) is a CD28 homologue, constitutively expressed on Tregs, which is rapidly translocated to the cell surface from the intracellular vesicle pool where it is stored. Following receptor engagement of naïve T-lymphocytes with antigen, the second activation signal is delivered through interaction of CD28 on the T-lymphocyte surface with CD80/CD86 expressed on antigen presenting cells (APCs). CTLA-4 acts as a crucial immune checkpoint by binding to CD80 and CD86, removing it from the APC membrane, thereby down-regulating and terminating T-lymphocyte-dependent responses after clearance of the pathogen. CTLA-4 insufficiency is inherited in an autosomal dominant fashion and clinical features include autoimmunity, enteropathy and lymphoproliferation. Two related diseases, lipopolysaccharide-responsive beige-like anchor protein (LRBA) deficiency and the differentially expressed in FDCP6 homolog (DEF6) deficiency have similar clinical patterns and all are characterized by reduced T-lymphocyte cell surface expression of CTLA-4. Gámez-Díaz and Seidel describe the signaling pathway, and use a novel scoring system, the immune deficiency and dysregulation score, to describe the clinical picture, contrasting it with other inborn errors of immunity.

Ikaros is a zinc finger transcription factor, encoded by *Ikaros Zinc Finger Protein 1* (*IKZF1*), which is required for early development, and late homeostasis, and function of T- and B-lymphocytes. Ikaros acts by regulating gene transcription through DNA binding, forming homo- or heterodimers with itself or other Ikaros Zinc Finger family transcription factor subunits. Boast et al. explore the role of Ikaros in lymphocyte development in mice, and look at human disease caused by mutations in *IKZF1*. Human disease is complex, as mutations in *IKZF1* can cause haplo-insufficient disease, which may be associated with incomplete penetrance or late-onset disease. It is typically characterized by symptoms of common variable immunodeficiency, namely recurrent respiratory tract infections associated with declining B-lymphocyte numbers and immunoglobulin levels. Dominant negative disease is less common, but more severe, and characterized by severe, early-onset combined immunodeficiency that is associated with development of haematological malignancies. More rarely, gene variants that affect dimerization in a haploinsufficient manner lead to a clinical presentation characterized by haematological cytopenias and malignancies, in which infection susceptibility does not significantly feature.

The phosphoinositide 3-kinase (PI3K) complex is constituted of a p110 catalytic subunit and a regulatory subunit. Autosomal dominant gain-of-function mutations in *PIK3CD*, which encodes the catalytic subunit p110δ cause type-1 Activated PI3-kinase-δ syndrome (APDS), whereas autosomal dominant loss-of-function mutations in one of the regulatory subunits; *PIK3R1* encoding the p85α, p55α and p50α, *PIK3R2* encoding p85β, or *PIK3R3* encoding p55γ, cause type-2 APDS. The hyperactivation of PI3Kδ leads to lymphoproliferation, bacterial and viral infections, auto-immunity and lymphoma. Thouenon et al. describe the biology and immunological characteristics as well as detailing the clinical presentation and treatment options in this disease entity.

Signal transducer and activator of transcription 3, encoded by *STAT3* is one of 7 STAT proteins and critical for regulation of cell survival, proliferation, differentiation, and effector function. STAT3 is activated *via* cytokines including IL-6, -10, -21, -23, and -27 and signals through the Janus (JAK) kinases, resulting in STAT3 phosphorylation. Whilst loss of function mutations in *STAT3* lead to impaired T_H_17 associated immune responses, gain of function mutations in *STAT3* lead to a clinical picture in which autoimmunity predominates, including early onset type I diabetes mellitus, autoimmune cytopenias, enteropathy and constitutional short stature, as well as lymphoproliferation (2). Vogel et al. review the biological, immunological and clinical characteristics of this disease.

Since the discovery of the *XIAP/BIRC4* gene and its subsequent association with *SH2D1A*-mutation negative X-linked lymphoproliferative disease (XLP-1), our understanding of this disease entity and the X-linked inhibitor of apoptosis (XIAP) itself has expanded. Patients with XIAP deficiency were initially identified as having similar symptoms to patients with XLP, particularly haemophagocytic lymphohistiocytosis (HLH). However it became apparent that XIAP-deficient patients typically have milder HLH disease, and do not develop lymphomas. Surprisingly, pathogenic variants in *XIAP* were also identified in patients with early-onset colitis where it is now recognized as one of the more common genetic causes of disease. We now know that HLH is less common in XIAP deficiency, which should more appropriately be considered a disorder of immune dysregulation and hyperinflammation. Initial results of haematopoietic transplantation were poor, but better results have been observed more recently using reduced intensity conditioning regimens. Mudde et al. give an overview of the biology and genetics of XIAP deficiency, and update clinical features and treatment strategies.

Many of the diseases described above result from disorders of lymphocyte development, control or disruption and loss of peripheral tolerance mechanisms. The final review in this series discusses autoimmune polyendocrinopathy-candidiasis-ectodermal dystrophy (APECED), caused by loss-of-function mutations in the autoimmune regulator (*AIRE*) gene. AIRE deficiency disrupts negative selection of T-lymphocytes in the thymus, causing a breakdown of central tolerance and enabling the peripheral escape of self-reactive T-lymphocytes. Ferre et al. review this disease, and outline treatment strategies.
